# Idiosyncratic hepatic toxicity in autosomal dominant polycystic kidney disease (ADPKD) patient in combined treatment with tolvaptan and amoxicillin/clavulanic acid: a case report

**DOI:** 10.1186/s12882-019-1612-7

**Published:** 2019-11-21

**Authors:** Angela Maria Pellegrino, Luigi Annicchiarico Petruzzelli, Eleonora Riccio, Antonio Pisani

**Affiliations:** Department of Public Health, Nephrology Unit, University of Naples “Federico II”, Naples, Italy

**Keywords:** ADPKD, Tolvaptan, Amoxicillin/clavulanic acid, Hepatic toxicity

## Abstract

**Background:**

Autosomal dominant polycystic kidney disease (ADPKD) is a hereditary disease characterized by the presence of renal cysts. Over time the expanding cysts lead to progressive renal failure. The use of tolvaptan, a V_2_-receptor antagonist, was recently approved in ADPKD patients. It was demonstrated that tolvaptan get slower decline in Kidney function compared with placebo. Idiosyncratic hepatic toxicity was described in patients receiving tolvaptan, with elevations in aminotransferases levels. We describe the first case reported in the literature in which hepatic toxicity is caused by the association of amoxicillin/clavulanic acid and tolvaptan.

**Case presentation:**

A 41 years old woman with diagnosis of ADPKD had been in treatment with tolvaptan for 16 weeks when an elevation of liver enzyme levels was detected. She had taken autonomously amoxicillin/clavulanic acid (in doses of 825/175 mg twice a day for 7 days) about 5 weeks before.

The timing of the event and the kind of hepatocellular injury could be attributed to the concomitance of medication of tolvaptan and amoxicillin/clavulanic acid.

**Conclusion:**

We highlight the need to careful monitor hepatic enzyme levels in order to recognize early hepatic side effects in ADPKD patients in treatment with tolvaptan and amoxicillin/clavulanic acid.

## Background

ADPKD is the most common hereditary kidney disorder, accounting for about 10% of patients on dialysis or living with a renal transplant [1]. It is characterized by progressive development and growth of cysts causing progressive kidney enlargement, destruction of renal parenchyma till to end stage renal disease (ESRD) [2–4].

For many years, the strategies employed to improve the quality of life and the patients’ life span were only based on lifestyle modifications, treatment of hypertension and renal and extra-renal complications.

In the last decade, advanced knowledge in genetics and molecular pathobiology of ADPKD focused on some aberrant molecular pathways involved in the pathogenesis of the disease [2, 3] leading to controlled clinical trials aimed to delay its progression.

Antidiuretic hormone arginine vasopressin promotes kidney-cyst cell proliferation and luminal fluid secretion by the up-regulation of its second messenger adenosine-3′,5′-cyclic monophosphate (cAMP) [5, 6].

Tolvaptan, a V_2_-receptor antagonist, has been shown to both reduce cyst burden and slow disease progression [6–8].

In the Tolvaptan Efficacy and Safety in Management of Autosomal Dominant Polycystic Kidney Disease and Its Outcomes (TEMPO) 3:4 trial, tolvaptan compared with placebo reduces kidney growth and the decline of estimated glomerular filtration rate (eGFR) over a 3 years period [9].

Adverse events were related predominantly to increased acquaresis (thirst, polyuria, nocturia and polydipsia). Elevations of liver enzyme levels were observed. More patients who received tolvaptan compared to those who received placebo (4.9% vs 1.2%) had reported levels of alanine aminotransferase (ALT) more than 3 times the upper limit of the normal range. This condition was defined as potentially clinical important. All these conditions became reversible after tolvaptan interruption or discontinuation. No persistent hepatic injury was reported [9].

Based on the results of the TEMPO 3:4 trial, the European Medicines Agency (EMA) approved in May 2015 the use of tolvaptan (JINARC®) for ADPKD [10, 11].

## Case report

A 41 years old woman with a medical history of hypertension and diagnosis of ADPKD was eligibile for treatment with tolvaptan. She was attending the Nephrology Center of the Federico II University of Naples. The patient had an average estimated glomerular filtration rate (eGFR) of 45.2 mL/min/1.73 m2 (CKD-EPI), CKD stage 3, serum creatinine was 1.42 mg/dl.

Jinarc® is to be administered twice daily in split dose regimens of 45 mg + 15 mg, 60 mg + 30 mg or 90 mg + 30 mg. The initial dose is 60 mg tolvaptan per day as a split-dose regimen of 45 mg + 15 mg. The initial dose is to be titrated upward to a split-dose regimen of 90 mg tolvaptan (60 mg + 30 mg) per day and then to a target split-dose regimen of 120 mg tolvaptan (90 mg + 30 mg) per day, if tolerated. Patients have to be maintained on the highest tolerable tolvaptan dose.

According to the criteria for use of tolvaptan in ADPKD set by the Agenzia Italiana del Farmaco (AIFA), blood testing for hepatic transaminases and bilirubin is required prior to initiation of Jinarc, continuing monthly for 18 months and at regular 3-monthly intervals thereafter to mitigate the risk of significant and/or irreversible liver injury. Concurrent monitoring for symptoms is also recommended.

The patient started with a split-dose regimen of 45 mg and 15 mg per day. The use of drug inhibiting the cytochrome P-450 enzyme CYP 3A4 was excluded.

After 28 days, the initial dose was titrated upward to a split-dose regimen of 90 mg tolvaptan (60 mg + 30 mg) per day (week 4). Physical examination, assessment of vital signs, blood and urine tests were performed before the increase. She reported no adverse effects, with the exception of effects related to acquaresis.

Dose was titrated to a target split-dose regimen of 120 mg tolvaptan (90 mg + 30 mg) per day after 28 days, according to patient tolerability and examinations (week 8).

**Fig. 1:**
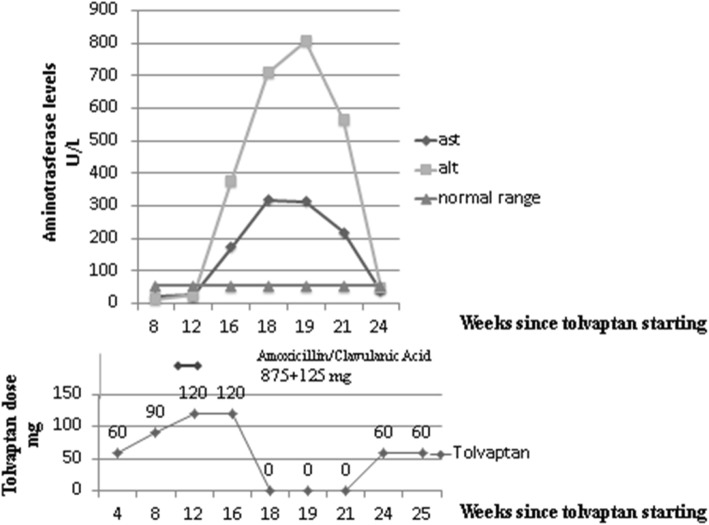
Amoxicillin/clavulanic acid and tolvaptan use in relation to the laboratory abnormalities

On week 12, blood testing was performed. Both transaminase levels (aspartate aminotransferase – AST, and ALT) had increased slightly although remaining below the upper limit of the normal range (Fig. 1). However, all other parameters were normal, therefore no further examination was performed and the treatment with the target dose (120 mg) was confirmed.

After 16 weeks of treatment, laboratory investigations showed a further increase of liver enzymes levels of more than three times the upper limit of the normal range (AST 172 U/L - normal range 0 to 34, ALT 378 U/L - normal range 0 to 55). There were no alterations in the other laboratory values and in the coagulation test, bilirubin total (BT) level was 0.25 mg/dl, alkaline phosphatase (ALP) 32 U/L (normal range 40 to 150), albumin 4.3 g/dl, γ-glutamyltransferase (GGT) 18 UI/ml, pseudocholinesterase (CHE) 8041 UI/ml, creatinine 1.8 mg/dl (vs 1.42 mg/dl before starting tolvaptan). Test for hepatitis A, B and C were negative. An abdominal ultrasonografy revealed no biliary obstruction, cancer or masses, but only multiple liver cysts, as reported in the precedent exams. The patient reported no symptoms; therefore, no further investigation about non hepatitis viral infections was performed. The patient had neither previous history of liver disease, nor of excessive alcohol intake, heart failure, shock or sepsis. She was carefully questioned about events or drug that could have led to liver injury. She only reported the use of antibiotics for fever and pharyngitis about 5 weeks before the start of transaminase increase (the antibiotic was taken at week 11). She had taken amoxicillin/clavulanic acid in doses of 825/175 mg twice a day for 1 week, without medical prescription.

As recommended, tolvaptan was immediately discontinued (week 16) [11].

Two weeks later (week 18), the laboratory report showed an additional increase of aminotransferases: AST 172 U/L, ALT 711 U/L (to > 12 times the upper limit of the normal range), followed 1 week later (week 19) by a further increase: AST 319 U/L, ALT 808 U/L. The blood test confirmed no more alterations.

Aminotransferase began to decrease after 2 more weeks (week 21): AST 220 U/L, ALT 565 U/L, and returned to normal levels after 2 months prior to presentation (week 24): AST 38 U/L, ALT 48 UI/L, (Fig. 1).

Following this, on week 24 she started again therapy with tolvaptan in doses of 45 mg and 15 mg, and no more elevation of aminotransferases was evidenced in the following monthly blood testing. They remained below the upper limit of the normal range.

## Discussion and conclusion

In patients with ADPKD, long-term treatment with tolvaptan is associated with idiosyncratic hepatic toxicity with elevations of ALT and AST and infrequent cases of concomitant elevations in BT. This injury is typically hepatocellular, occurs between 3 and 18 months after starting tolvaptan, and resolves itself within 4 months after stopping the drug 12 [11, 12].

Moreover, amoxicillin/clavulanic acid is a relatively frequent cause of hepatic injury, which is characterized by a hepatocellular/cholestatic reaction and often by evidence of immunoallergy. But in some cases, especially in patients under 45 years old, it can occur together with hepatocellular injury. Typically, the latency of presentation is about 1 or 2 months after the start of treatment [13].

In our case report, the likelihood of drug-induced liver disease is supported by several pieces of evidence: the temporal association, the resolution after the withdrawal of the medications, no other possible diagnosis. Temporal association could prove drug-induced hepatotoxicity for both of the drugs (about 1 or 2 months after the start of amoxicillin/clavulanic acid, between 3 and 18 months after starting tolvaptan). As a matter of fact, in this case report, liver injury classified according to laboratory parameters as acute hepatocellular injury, occurred about 3 months after the beginning of the treatment with tolvaptan and 5 weeks after the therapy with amoxicillin/clavulanic acid. Moreover, the liver disease resolved itself after the withdrawal of the medications and there is no other possible diagnosis.

There is another case reported in literature that describes an elevation of AST, ALT and BT after a single dose of amoxicillin/clavulanic acid of 8 g between 2 and 3 months before liver injury [14]. But in this case report, the timing of the event and the single dose do not appear to justify the amoxicillin/clavulanic acid role in causing hepatotoxicity. In fact, blinded consensus judged the adverse effect to be probably related to tolvaptan [14].

Furthermore in our case it is impossible to state whether the hepatotoxicity was related to tolvaptan, amoxicillin/clavulanic, or their combined use. As a matter of fact, it could just be a simple independent reaction to either tolvaptan or amoxicillin/clavulanic acid, rather than their synergy.

On the other hand, we cannot exclude the causality of tolvaptan–induced hepatotoxicity, even if its readministration did not result in recurrence. Evidences show that approximately half of the subjects were able to tolerate the drug when it was reintroduced, after hepatic injury, suggesting that a form of adaptation or drug tolerance occurs. However, the other half experienced rapid ALT elevations upon re-exposure and tolvaptan treatment had to be permanently withdrawn [12]. We decided to reintroduce tolvaptan treatment with starting dose (split dose regimens of 45 mg + 15 mg), and to not titrate to a target dose of 120 mg to avoid the risk of significant and/or irreversible liver injury. Even if no association with dose was found, the reasoning to use the lowest dosage of tolvaptan is due to the fact that additional research are necessary and has been initiated to further investigate the role of dose and exposure on the risk of hepatic injury [12].

In conclusion, this represents the first case reporting hepatic toxicity in an ADPKD patient during the combined therapy of amoxicillin/clavulanic acid and tolvaptan; therefore, more caution should be used when prescribing amoxicillin-clavulanic acid in ADPKD patients treated with tolvaptan.

## Data Availability

The datasets used during the current study are available from the corresponding author on reasonable request.

## Abbreviations

ADPKD: Autosomal dominant polycystic kidney disease
ALP: Alkaline phosphatase
ALT: Alanine aminotransferase
AST: Aspartate aminotransferase
BT: Bilirubin total
cAMP: Adenosine-3′,5′-cyclic monophosphate
CHE: Pseudocholinesterase
eGFR: Estimated glomerular filtration rate
EMA: European Medicines Agency
GGT: γ-glutamyltransferase
TEMPO: Tolvaptan Efficacy and Safety in Management of Autosomal Dominant Polycystic Kidney Disease and Its Outcomes
